# Mammalian malaria: Remembering the Alamo

**DOI:** 10.1080/21505594.2020.1793489

**Published:** 2020-07-27

**Authors:** Colin J. Sutherland

**Affiliations:** Department of Infection Biology, Faculty of Infectious & Tropical Diseases, London School of Hygiene & Tropical Medicine, London, UK

**Keywords:** *Plasmodium*, inflammatory response, antibody, endothelial activation, pediatric severe malaria

Much attention has been directed toward a recent slowing of progress in malaria control worldwide, with particular emphasis on growing evidence which suggests that the twin pillars of malaria reduction – chemotherapy and anti-mosquito measures – are both less effective than previously. This special issue of *Virulence* gathers papers with a different perspective – understanding the response of the mammalian host to infection with the *Plasmodium* parasite. Taken together, this collection of writing is based on an implicit understanding of the human body as a fortress against invasion by the marauding parasite. Innate defenses such as fever, phagocytosis, and systemic inflammation, and the pathogen-elicited adaptive responses of cellular immunity and antibody are the walls, ramparts, boiling oil, and fortified gates that protect us from malaria, as from other infections. In this series, our panel of expert authors examine how these defenses work, the parasite’s strategies for eluding them, what happens when they fail and progression to severe disease occurs, and how biomarkers of the body’s defense systems can be valuable diagnostic indicators of morbidity and prognosis. This knowledge is of great value in the continued effort to develop improvements in the management of severe malaria, by directing defenses down beneficial pathways to minimize pathology, and in the design of effective vaccines against *Plasmodium* parasites.

## Decision making in the host microvasculature

The endothelium of the microvasculature of the human host is a crucial tissue in *P. falciparum* infection. Parasite-derived ligands on the surface of the infected erythrocyte mediate receptor binding, enabling sequestration of maturing blood-stage asexual parasites away from splenic filtration. This binding results in endothelial activation and damage to the vessels, and this injury is a key feature of severe life-threatening malaria (SM). This is an example of harmful host responses which can lead to increased pathology. However, only a minority of infections progress to SM, and so the host is usually able to manage this interaction. Clara Erice and Kevin Kain [1] explore the processes that lead to these very different outcomes and consider ways to exploit any host molecules indicative of very early endothelial responses as biomarkers for risk of progression to SM. Patel, Dunican, and Cunnington [2] also train a microscope on features of disease progression, but concentrate on pediatric SM at admission – what features of the illness can be utilized as biomarkers to assist in reaching a rapid prognosis and permit optimal management of each child on a case-by-case basis? These considerations could lead to an understanding of how to intervene in such a way as to guide immune responses in malaria patients toward reduced pathology and so better outcomes.

## Inflammatory immunopathology and the marshaling of antibody defenses

Inflammation is a feature of illness caused by all *Plasmodium* species, and has been examined closely in experimental rodent infections, where its potential to contribute to immunopathology and SM is well known. Lisa Drewry and John Harty [3] guide us through a substantial literature elucidating the symphonic action of innate, cellular, and antibody responses of the immune system to parasite infection, the result being a crescendo of inflammation and potential risk of pathology if IFN-γ and Th1 T-cells are conducting the orchestra. In contrast, TGF-β and IL-10 can guide the immune players to an anti-inflammatory state, with a lower risk of pathology. The authors look closely at the role of regulatory T-cells in these events. Hahn, Pepper and Liles [4] rather point us to the role of B-cells, presenting new data on the signaling role of the type III interferon IFNλ, highlighting its suppression of the acute antibody response and thus impedence of parasite clearance early in infection. Allison Bucşan and Kim Williamson [5] develop the B-cell theme further, asking difficult questions concerning the obstacles to the development of effective malaria vaccines that elicit a robust humoral attack on the many parasite protein antigens involved in erythrocyte invasion. Their thesis is that vaccine design should incorporate our rapidly developing understanding of the action of parasite nucleic acids, haemozoin, and phospholipid, continually released by cycles of schizogony, in initiating signaling pathways to elicit antibody responses. Such signaling early in infection may be crucial in determining outcomes and provide the prognostic markers sought by Patel, Dunican, and Cunnington [2].

## Oranges are not the only fruit

SM can be the result of infection with *Plasmodium* species other than *P. falciparum*. Anupkumar Anvikar, Sam Wassmer, and colleagues [6] present recent findings from severe malaria cases in India caused by infection with *P. vivax*, the most common causative agent for malaria on the sub-continent. In the study region, Gujarat, severe malaria hospital admissions from vivax malaria outnumber those with *P. falciparum* infections. Distinct features of SM in *P. vivax* disease are discussed, such as jaundice, and the observed frequency of severe disease in adults, perhaps indicating a waning of malaria immunity in this population. This paper is an example of “real world” immunology in settings where infections can be complex mixes of multiple parasite species simultaneously, or where a single individual can experience alternating bouts of malaria caused by different species.

## Controlling malaria requires diverse approaches

This collection of papers reminds us that, along with the need to target mosquito transmission and the necessity of providing rapid access to effective malaria drugs, malaria control strategies must also enlist the defensive capabilities of the human immune system – do not forget the Alamo. This is not an easy task, as illustrated by our dearth of effective vaccines (only one has been registered for use in humans to date, Mosquirix™), our lack of diagnostic markers for risk of complicated disease and our currently imperfect understanding of how to minimize harmful pathology in severe malaria. The work described herein is therefore presented as an indication of progress using a number of promising approaches, each of them a reason for optimism that this task may yet be accomplished.
